# Analysis of the Spread and Evolution of COVID-19 Mutations in Ecuador Using Open Data

**DOI:** 10.3390/life14060735

**Published:** 2024-06-07

**Authors:** Cesar Guevara, Dennys Coronel, Byron Salazar, Jorge Salazar, Hugo Arias-Flores

**Affiliations:** 1 Centro de Mecatrónica y Sistemas Interactivos—MIST, Universidad Tecnológica Indoamérica, Quito 170301, Ecuador; dcoronel1@indoamerica.edu.ec (D.C.); hugoarias@uti.edu.ec (H.A.-F.); 2 Neurosurgery Department, Hospital de las Fuerzas Armadas HE-1, Quito 170136, Ecuador; bsalazar@hmetro.med.ec; 3 Neurosurgery Department, Metropolitano Hospital, Quito 170521, Ecuador; jsalazarf@hmetro.med.ec

**Keywords:** COVID-19, virus spread, Crisp-DM methodology, virus genome, epidemiology

## Abstract

Currently, the analyses of and prediction using COVID-19-related data extracted from patient information repositories compiled by hospitals and health organizations are of paramount importance. These efforts significantly contribute to vaccine development and the formulation of contingency techniques, providing essential tools to prevent resurgence and to effectively manage the spread of the disease. In this context, the present research focuses on analyzing the biological information of the SARS-CoV-2 viral gene sequences and the clinical data of COVID-19-affected patients using publicly accessible data from Ecuador. This involves considering variables such as age, gender, and geographical location to understand the evolution of mutations and their distributions across Ecuadorian provinces. The Cross-Industry Standard Process for Data Mining (CRISP-DM) methodology is applied for data analysis. Various data preprocessing and statistical analysis techniques are employed, including Pearson correlation, the chi-square test, and analysis of variance (ANOVA). Statistical diagrams and charts are used to facilitate a better visualization of the results. The results illuminate the genetic diversity of the virus and its correlation with clinical variables, offering a comprehensive understanding of the dynamics of COVID-19 spread in Ecuador. Critical variables influencing population vulnerability are highlighted, and the findings underscore the significance of mutation monitoring and indicate a need for global expansion of the research area.

## 1. Introduction

Undoubtedly, the analysis of the spread of communicable diseases has become an extremely promising area of research. This is attributed to its significant contributions in recent years to the development of vaccines, prevention techniques, and health response plans [1,2]. In the last two decades, we have witnessed the spread of numerous communicable diseases among different countries. Recent studies, including Prabu’s work [3], elaborate that these diseases have been directly transmitted by bacteria, viruses, and other pathogens.

According to the International Federation of Red Cross and Red Crescent Societies (IFRC) and the World Health Organization (WHO), the most contagious diseases globally include tropical diseases such as tuberculosis, malaria, coronavirus, dengue, hepatitis, measles, and HIV/AIDS [4]. These diseases pose significant global public health challenges due to their capacity to spread and their impact on the affected populations. A notable instance of the spread of communicable diseases is the MERS-CoV virus, which has caused severe respiratory infections in more than 2468 people, resulting in over 851 deaths in 27 countries since 2012 [5].

In 2009, the swine flu virus (H1N1) emerged, spreading to 214 countries and causing more than 18,449 confirmed deaths, as reported by the WHO [6]. However, this was not the only case of this nature during the aforementioned period. Between 2003 and 2019, the avian influenza virus (H5N1) emerged, infecting 861 people worldwide [7]. Another virus of significant spread and relevance was the severe acute respiratory syndrome (SARS) pandemic, which originated at the end of 2002 and extended to 29 countries. SARS caused 8096 cases of infection and resulted in 774 deaths. More recently, the COVID-19 virus, likely originating in Wuhan, China, in 2019, caused a global epidemic that has severely affected numerous countries [8]. According to the WHO [9], COVID-19 has affected 770 million people, with 6 million lives lost due to this disease.

In the case of Ecuador, more than one million people have been diagnosed with COVID-19, and over 36,000 people have lost their lives [10]. However, it is crucial to note that a significant vaccination campaign has been conducted in the country. According to data from the Ministry of Health of Ecuador [11], nearly 39 million doses of vaccines have been administered to the population.

Research in this field is of vital importance, especially in the context of the coronavirus disease. Disease spread is a matter of the utmost significance due to the unpredictable evolution and mutation of viruses globally [12]. This is particularly relevant in Ecuador, a country characterized by unique climatic diversity, which could be conducive to the development of various mutations.

A relevant work on this topic is Rui Wang’s study [13], which identified and analyzed the positions, frequencies, and encoded proteins of SARS-CoV-2 mutations globally. The primary objective of this study was to isolate the SARS-CoV-2 genome and quantify the number of mutations present using the genotyping technique. The results were considered satisfactory, as they identified a total of 13,402 unique mutations. Additionally, the study revealed that 51.4% of the SARS-CoV-2 mutations corresponded to the C⟶T type.

Wang [14] identified rapidly proliferating mutations in the receptor-binding domain (RBD) and analyzed the evolutionary trend of SARS-CoV-2. The primary goal was to examine a genomic dataset of SARS-CoV-2 recorded in the Mutation Tracker using a deep learning method. The results are highly encouraging, highlighting 6945 unique mutations and 2,194,305 non-unique mutations in the SARS-CoV-2 S gene worldwide. Furthermore, the authors determined that the majority of mutations in SARS-CoV-2 corresponded to the A⟶G, C⟶T, and T⟶C types. They also indicated that approximately 70% of these mutations can weaken the efficacy of known antibodies.

Thanh [15] undertook a comprehensive analysis of genomic mutations in the coding regions of SARS-CoV-2, exploring the potential secondary structure of the resulting proteins. The central objective of this study was to assess all point mutations recorded to date in SARS-CoV-2. This study further identified different mutation patterns using various deep-learning models. A total of 3089 mutations were found in the S protein of SARS-CoV-2. Lucy Van Dorp [16] analyzed mutations associated with SARS-CoV-2 virus transmission with the aim of quantifying the number of offspring that inherited a specific allele compared to those who did not. The phylogenetic index was employed for the data analysis in this study. The results revealed a total of 12,706 C⟶U-type mutations. However, the study concluded that none of these mutations were associated with a significant increase in virus transmission.

Pachetti et al. [17] conducted an analysis and evaluation of the distribution of SARS-CoV-2 mutations in various geographical areas (Asia, Oceania, Europe, and North America) using the Clustal Omega method. This study relied on randomly collected data from the GISAID database. The work produced significant findings, identifying a total of 14,408 mutations in the P to L proteins. Furthermore, the authors demonstrated that some of these mutations could lead to resistance to certain drugs.

Rozhgar [18] identified and analyzed the genomic mutations of SARS-CoV-2. The primary objective was to determine the most common SARS-CoV-2 mutations using bioinformatics programs. The study analyzed 95 complete SARS-CoV-2 genome sequences available at the GenBank National Microbiology Data Center (NMDC). The results showed 116 mutations corresponding to the ORF1ab gene, ORF8, and the N gene. Ahmad [19] analyzed the whole-genome mutations of SARS-CoV-2. The primary objective of this study was to determine the possible mutations and evolution of COVID-19. The study utilized BioEdit software version 7.2 to conduct genomic alignments and determined that there were 596 mutations across all genes.

Lastly, Abdel-Rahman [20] analyzed the sequential mutations present in the SARS-CoV-2 genome and determined the various mutation patterns manifested in infected Egyptian patients. The author utilized the Pangolin and Nextstrain lineage declassification methods with the primary objective of determining the optimal classification of SARS-CoV-2 genomes. The results revealed the existence of a total of 1115 unique mutations. Further, approximately 60.5% of these mutations were located in the ORF1ab polyprotein.

Thus, the central objective of the present investigation is to analyze both the biological information of viral variants and the clinical data of patients infected with COVID-19 in Ecuador. This analysis encompasses variables such as age, gender, and geographic location, among others. The goal is to identify the most relevant variables and to comprehend the evolution of virus mutations, as well as their geographic distributions in various provinces of Ecuador. Through these data analyses, we aim to pinpoint the most vulnerable population groups based on their clinical characteristics, such as age, gender, and geographic location, concerning the diverse mutations and variants of SARS-CoV-2. This approach will foster a more profound understanding of the disease dynamics and enable more informed decision-making in terms of public health.

This paper is structured as follows. Section 2 details the methods and materials used to conduct the research. Section 3 focuses on the phases of data preprocessing and the application of technological tools to the information. Section 4 discusses the analysis of the data obtained, encompassing information related to variants of viruses, as well as patient profiles extracted from the database. The discussion is further enriched by incorporating perspectives and findings from other authors in the field. Finally, in Section 5, conclusions derived from the findings are presented, and possible directions for future work are discussed.

## 2. Methods and Materials

In this section, detailed information on the materials, including the database used, and the methods applied for data analysis in the development of this study is presented.

### 2.1. Materials

#### COVID-19 Database—GISAID Ecuador

The COVID-19 database in Ecuador was compiled through EpiFlu™, an initiative of GISAID [21]. This dataset consists of a total of 8992 records and 13 attributes. In total, 1 attribute contains genetic information (virus genetic sequences), and the remaining 12 attributes include medical information related to patients, as presented in Table 1. This database contains different variants of SARS-CoV-2 (Omicron, Delta, Epsilon, Gamma, Lambda, and Alpha). However, the Beta variant is not included because the GISAID website lacks Ecuadorian records for this variant.

In Table 1, the virus code row has the GRA code, which, in the context of COVID-19, refers to the timeline of virus variants. GRA is an abbreviation that the World Health Organization (WHO) uses to identify Variants of Interest (VOIs). After processing the protein chain data, a new database was generated that incorporated information on the 150 amino acids frequently mutated in the SARS-CoV-2, along with the number of mutations associated with each of them.

## 3. Data Processing

Data preprocessing is a crucial phase as the efficiency of preprocessing greatly influences the quality of the final results. Data processing was divided into four distinct phases based on the CRISP-DM methodology, in which a series of techniques and transformations were applied to the data to clean, organize, and prepare them for further analysis.

### 3.1. CRISP-DM Methodology

**Figure 1:**
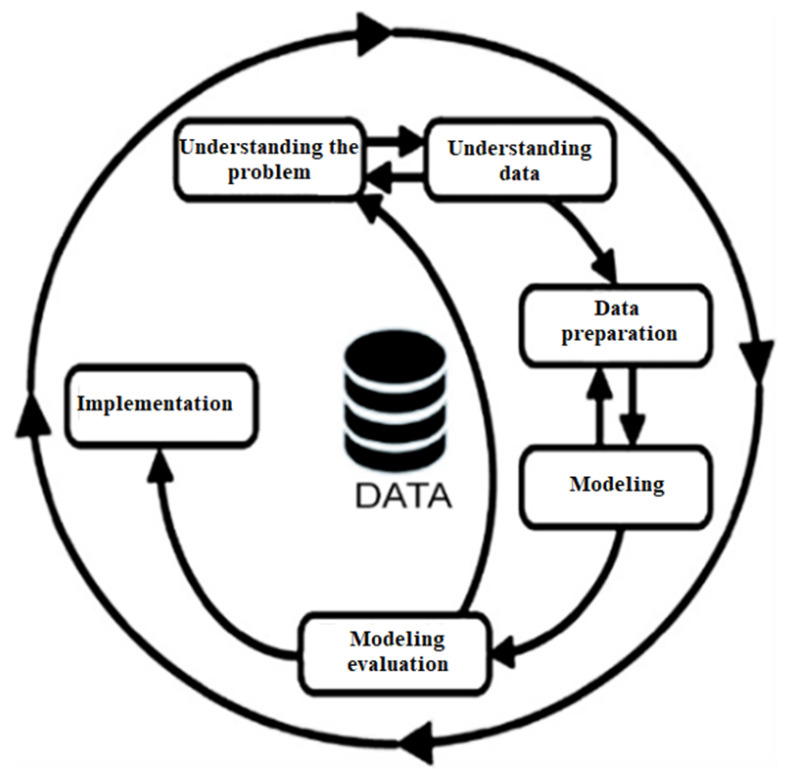
Stages of the Crisp-DM methodology [23].

The Cross-Industry Standard Process for Data Mining (CRISP-DM) methodology is one of the most widely used for the development of data mining projects, as it offers a cyclic approach to project management. This methodology allows for a structured life cycle, facilitating the understanding and efficient management of each stage of the data mining process [22]. Figure 1 illustrates each stage, starting with problem understanding, followed by data understanding, data preparation, modeling, model evaluation, and implementation.

The CRISP-DM methodology consists of six fundamental stages. It begins with understanding the problem, where the problem is identified, the project objectives are defined, and the current state is evaluated. Subsequently, in the data understanding phase, data are collected and explored to understand their meanings and properties. The data preparation stage focuses on cleaning, transforming, and creating indicators from existing data. In the modeling stage, the most appropriate technique is chosen, the model parameters are adjusted, and its performance is evaluated. The evaluation stage determines the model’s quality through statistical metrics and comparisons with previously established objectives to ensure that it meets the expectations of the project. A satisfactory evaluation of the trained model is followed by its implementation, during which a specific infrastructure for data processing is configured [23].

### 3.2. Preprocessing

For each phase of data processing, we utilized the server of the Institute of Mathematical Sciences (ICMAT), known as the LOVELACE Cluster. This server comprises 32 general computing nodes, one node equipped with Xeon Phi processors, two nodes with graphics processing units (Tesla GPUs), and three nodes with high RAM capacity.

#### 3.2.1. Phase 1—Integration and Data Collection

In this phase, the COVID-19 database from Ecuador was downloaded from the GISAID platform [21]. This database was divided into two parts: the first in a FASTA format containing information on the protein chain, date, and patient code, among other relevant details. The second part comprised patient medical data in .CSV files containing epidemiological information, such as age, gender, patient ID, location, and variant.

#### 3.2.2. Phase 2—Data Selection and Cleaning

During this phase, data problems were identified and corrected to ensure the accuracy and reliability of the information. For this procedure, an algorithm called “DataClean” was developed and created using the high-level Python programming language. This algorithm utilizes multiple data management libraries such as Pandas, Numpy, and Matplotlib. The algorithm eliminates duplicate data, outliers, and data containing errors. Additionally, it cleans the information on each variable by eliminating line breaks and unknown characters. In this phase, irrelevant variables for the study were also removed, such as host, as all those infected with the virus are human; originating_lab due to incoherent, incomplete, and imprecise information; authors, as it only shows who collected the sample; and originVariant because of its incomplete information that does not contribute to the research.

Table 2 shows the Pearson correlation coefficients between the variables.

Table 3 presents the values of the chi-square statistic between the variables.

Table 4 displays the values of the ANOVA statistic, with F-statistics representing the tool used to analyze significant differences between variables.

#### 3.2.3. Phase 3—Data Transformation

In this phase, the data were prepared for further analysis, including the standardization of data (age and gender). The categorization of the age variable was carried out, representing 20 groups of data in 5-year intervals: Group 1 (0–5 years), Group 2 (6–10 years), Group 3 (11–15 years), Group 4 (16–20 years), Group 5 (21–25 years), Group 6 (26–30 years), Group 7 (31–35 years), Group 8 (36–40 years), Group 9 (41–45 years), Group 10 (46–50 years), Group 11 (51–55 years), Group 12 (56–60 years), Group 13 (61–65 years), Group 14 (66–70 years), Group 15 (71–75 years), Group 16 (76–80 years), Group 17 (81–85 years), Group 18 (86–90 years), Group 19 (91–95 years), and Group 20 (96–over 100 years). In this data preparation phase, we decided to categorize the age variable into 5-year groups to better structure and organize the information for further analysis. This choice allows for a detailed representation of the age distribution in the sample and facilitates the identification of specific patterns and trends in different population segments. Furthermore, the division into small intervals provides a more accurate view of how age can influence the results, enabling a more precise interpretation of the findings.

The gender variable was transformed into two numerical categories (0: male and 1: female) to apply variable selection techniques, such as ANOVA [24] and chi-square [25], which do not allow for the analysis of categorical data. Protein chain standardization was carried out, where genetic sequence alignment was performed using the Multiple Alignment Fast Fourier Transform (MAFFT) [26] version 7 system with the progressive Fast Fourier Transform method and the iterative refinement method (FFT-NS-2) [27]. The primary purpose of this phase was to align the viral gene sequences of each patient to a reference sequence of uniform length (29,904 nucleotides) for all. This process was executed using an algorithm developed in Python. Subsequently, the dataset of aligned gene sequences of the same size was transformed into amino acid chains using the EigenMS [28] and LibMUSCLE [29] libraries.

Lastly, the resulting amino acid chain was utilized to identify the number of mutations present in each record and compared to the patient’s COVID-19 sample in the early stages of infection. This task was executed with a Python algorithm using the Biopython library and Pandas. The primary objective of this algorithm was to identify the various mutations present in each amino acid. To detect these mutations, changes in amino acids at specific positions in the sequence were analyzed in comparison to other sequences. As a result, a database was generated detailing the number of mutations affecting each amino acid.

#### 3.2.4. Phase 4—Data Integration

In this final phase, an algorithm was developed to integrate the patient’s epidemiological database with the amino acid database and the number of detected mutations of the virus in Phase 3. For this data integration, a Python 3.11.3 algorithm was created to merge the two datasets based on the patient’s ID. Through this data fusion, a cohesive dataset was established, enabling a comprehensive and unified analysis.

## 4. COVID-19 Data Analysis and Results

This section presents data analysis related to COVID-19 patients in Ecuador post-data preprocessing. To conduct this analysis, Python, Matplotlib, Geopandas, and Seaborn libraries were employed to visualize the processed information. These libraries facilitated the generation of statistical diagrams and variable correlations, thereby easing the examination of each graph.

Table 5 presents the total number and infection rate of SARS-CoV-2 variants in different provinces of Ecuador. Table 6 illustrates the correlation between the provinces most impacted by various variants of SARS-CoV-2, along with the corresponding infection percentages. The data highlighted Pichincha, Guayas, and, to a lesser extent, Chimborazo as the provinces experiencing the highest infection percentages linked to different variants of SARS-CoV-2.

**Figure 2:**
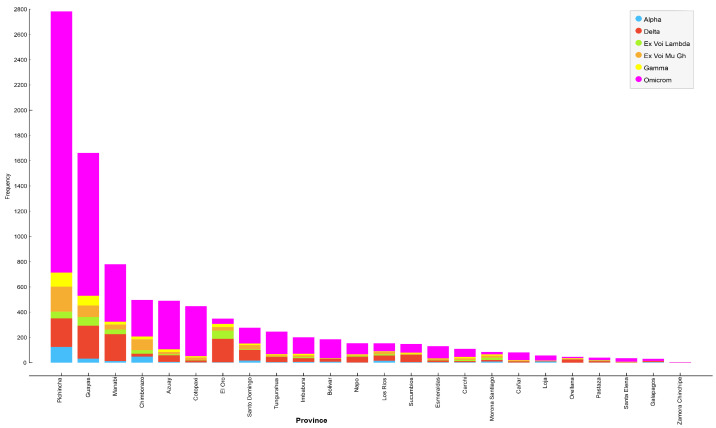
SARS-CoV-2 variants by province in Ecuador.

Figure 2 depicts the distribution of various SARS-CoV-2 variants across the provinces of Ecuador. Provinces with the six highest infection rates include Pichincha, Guayas, Manabí, Chimborazo, Azuay, and Cotopaxi. Moreover, the most prevalent variant in each province is Omicron, followed by Delta, Mu Gh, Gamma, Lambda, and, lastly, the Alpha variant (Table 6).

**Figure 3:**
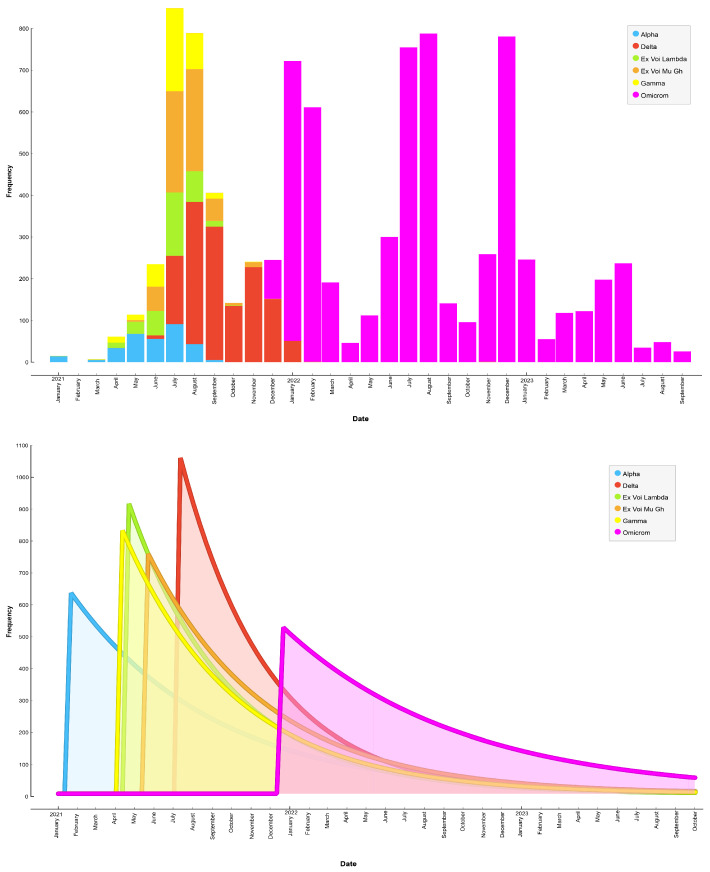
Chronology of the spread of SARS-CoV-2 variants.

Figure 3 presents a plot illustrating the chronology of SARS-CoV-2 contagion by variant from January 2021 to October 2023. The results showed that the Alpha variant was predominant until July 2021, followed by the Delta variant from July 2021 to January 2021. However, the variant that consistently remained predominant in Ecuador is Omicron, which began spreading from December 2021 to October 2023.

**Figure 4:**
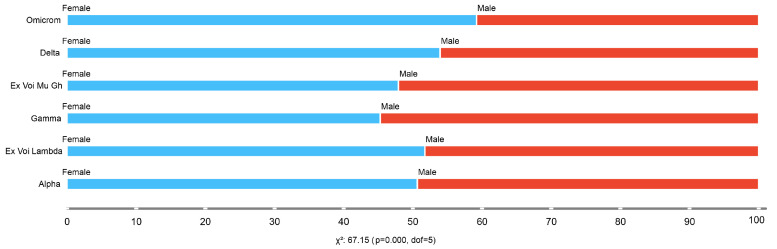
SARS-CoV-2 variants by patient gender.

Figure 4 illustrates the quantitative distribution of SARS-CoV-2 variants according to patient gender. We observed that female patients were highly affected by the Omicron variant, representing 69.17% of the cases, followed by the Delta variant, which was detected in 14.88% of cases. Conversely, male patients were preferrently affected by the Omicron variant, constituting 61.72% of the cases, followed by the Delta variant in 16.46% of cases. These findings underscore significant differences in SARS-CoV-2 variant prevalence between genders.

**Figure 5:**
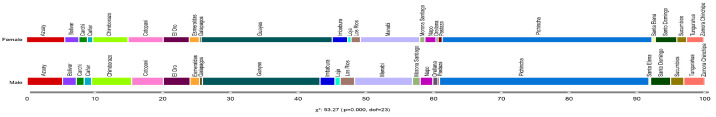
Infected persons by gender and province.

Figure 5 presents a quantitative distribution concerning individuals infected by SARS-CoV-2, categorized by gender and province. It illustrates that female COVID-19 patients were observed predominately in the provinces of Pichincha (30.97%), Guayas (19.36%), and Manabí (8.75%). Similarly, male patients showed a high incidence in the provinces of Pichincha (30.91%), Guayas (17.36%), and Manabí (8.55%). These findings indicate that the provinces of Pichincha, Guayas, and Manabí were the most affected by the COVID-19 pandemic, significantly impacting both female and male patients.

**Figure 6:**
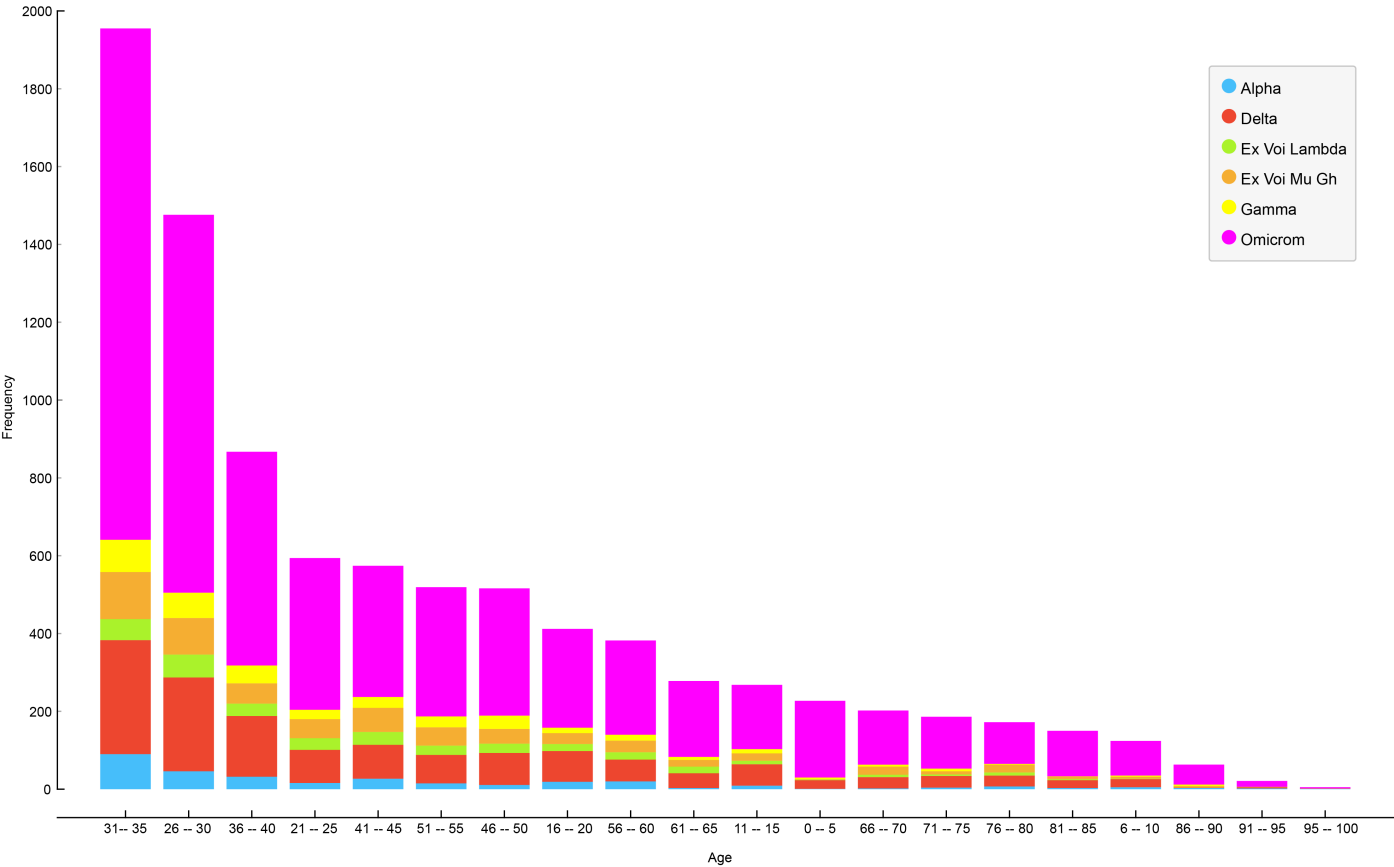
Infected patients by age and SARS-CoV-2 variant.

Figure 6 illustrates the age distribution of patients for each variant of SARS-CoV-2 in Ecuador, revealing that the most affected group consisted of patients between 31 and 35 years of age, followed by patients aged 26–30, and, in third place, patients aged 36–40.

**Figure 7:**
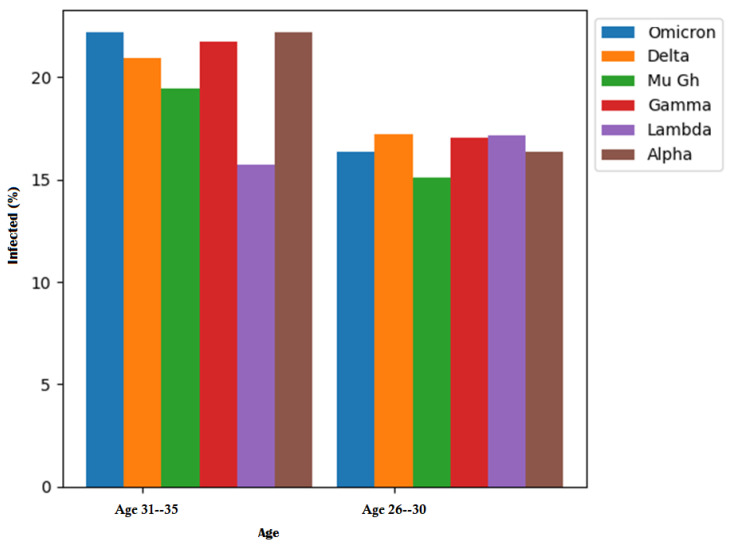
Higher percentage of infected patients by age and variant.

Figure 7 illustrates the primary distribution of infected patients by age and SARS-CoV-2 variant. Table 7 provides a detailed breakdown of the ages affected by each COVID-19 variant. In the case of individuals aged 31–35 years, the Omicron variant accounted for 22.17%, the Delta variant for 20.93%, the Mu Gh variant for 19.45%, and the Gamma variant for 21.73% of cases. The Lambda variant showed a higher impact on patients aged 26–30 years, with 17.15%, while the Alpha variant affected 22.17% of patients.

### Discussion

The analysis of the biological information of the virus specifically focused on the spike protein, which, as observed in prior studies, such as those by Wang [14] and Thanh [15], exhibits the highest number of mutations. As shown in Table 8, the majority of amino acids with two mutations belong to the spike protein (S) chain. Mutations in this protein chain may impact transmissibility, the ability to evade the immune system, and vaccine efficacy. Notably, 98% of amino acids generating two mutations are associated with the spike protein, while the remaining 2% lack a defined protein chain.

The “Mutations” column delineates the type of genetic change occurring at a specific location in the genomic sequence. The “Protein” column identifies the protein linked to that mutation. The “Position” column specifies the precise location within the genomic sequence where the mutation is recorded. The “Original Sequence” column displays the reference genetic sequence at the affected location, while the “Mutated Sequence” column illustrates the genetic alteration. In instances where no specific protein is assigned to the sequences, informations on the original sequence and the mutated sequence are unavailable.

Table 9 displays the amino acids most significantly affected by mutations, offering crucial details such as the associated protein chain, the specific position within the sequence, the original amino acid sequence, and the resulting amino acid sequence following the mutation. The findings reveal that the predominant portion of amino acids affected by mutations is within the spike protein (S), accounting for 68.29% of cases. Additionally, 14.63% of intances are associated with the nucleocapsid (N) protein, 14.63% with the non-structural protein (nsp2), and 2.45% with the non-structural protein (nsp1).

Table 10 illustrates the amino acids that harbor two mutations in various variants. The most noteworthy are E484A and P681R. Amino acid E484A has been linked to a decrease in antibody effectiveness. Furthermore, the presence of the P681R amino acid in the spike protein of the virus may impact its ability to enter human cells.

Table 11 presents a comprehensive overview of the amino acids that have undergone mutation in various variants, specifying the protein chain to which they belong. The most frequently altered amino acids among variants are D614G, N501Y, and P681H. Amino acid D614G has been linked to enhanced virus transmission capability. Moreover, amino acid N501Y has been associated with a potential increase in virus binding to host cells. Lastly, amino acid P681H, similar to the P681R mutation, affects virus infectivity by influencing its ability to enter human cells. These amino acids, as listed in Table 11, are particularly noteworthy due to their potential impact on transmission dynamics and the virus’s interaction with host cells.

The results obtained from the analysis of COVID-19 in Ecuador exhibit similarities with those reported by Wang [14] and Thanh [15], who indicated that the majority of mutations are associated with the spike protein (S). Conversely, Rozhgar’s findings [18] align with our results, establishing that the affected protein chain includes nucleocapsid (N). Furthermore, Pachetti et al.’s study [16], similar to our study, identified a set of affected amino acids, specifically those located in the protein sequence from “P” to “L”. Notably, mutations P1000L and P2046L within this sequence were prominent, being present in a high percentage of infected patients across the Omicron, Delta, Mu GH, and Alpha variants. These specific amino acids (P1000L and P2046L) underwent two significant mutations, emphasizing their association with the aforementioned virus variants and their recurrent presence in a substantial number of infected patients.

The literature review did not identify any comparable studies with the epidemiological data of COVID-19 from Ecuador.

## 5. Conclusions and Future Work

In this study, an in-depth analysis of COVID-19 data from Ecuador was conducted. This involved examining both the biological information from the viral variants and patient-related epidemiological data obtained through the GISAID initiative. Throughout the research, diverse data preprocessing and statistical analysis techniques were applied, including Pearson’s correlation, the chi-square test, and analysis of variance (ANOVA). Furthermore, statistical diagrams and graphs were utilized to enhance the visualization of the results.

The CRISP-DM methodology is utilized in numerous data mining projects yet exhibits notable limitations. First, it primarily focuses on the initial stages of a project, as a comprehensive grasp of the research domain is imperative for progressing to subsequent phases. Second, it lacks a robust emphasis on validating the obtained results. However, a vital advantage of the CRISP-DM methodology is its adaptability to meet the specific requirements of each project. This flexibility facilitates the integration of additional techniques, such as protein alignment and the transformation of proteins into amino acid chains, along with strategies to identify the most relevant mutations in our study. Consequently, this integration enhances both the procedural aspects and the outcomes obtained.

Clearly, this study generated significant findings by analyzing the geographic distribution of COVID-19-related variables in various provinces of Ecuador. We observed that the Omicron variant was more prevalent in a large part of the Ecuadorian territory, closely followed by the Delta variant, while the Lambda variant was present in some specific regions. In this context, we found that the Omicron, Delta, and Lambda variants affected more than 50% of female patients, while the Mu GH and Gamma variants affected more than 50% of male patients. A higher incidence of COVID-19 was observed in female patients in the provinces of Pichincha with 30.97%, Guayas with 19.36%, and Manabí with 8.75% of cases.

A high incidence among male patients was reported in the provinces of Pichincha (30.91%), Guayas (17.36%), and Manabí (8.55%), indicating that these provinces were the most affected by the disease. The findings further revealed that the age group of 26 to 35 years was the most affected by all variants, with the Delta variant being more noticeable in patients aged 31 to 35 years, while the Mu GH variant had a relevant impact on patients aged 31 to 35 years and 41 to 45 years. We also found that from January 2021 to the end of November 2021, the predominant variants in infections were Mu GH, Gamma, and Delta. However, from the beginning of December 2021 until 2023, the most prevalent variant was Omicron.

In addition, highly relevant genomic information highlighting the relationship between variants and amino acids was discovered, including the fact that amino acids associated with the S protein, nucleocapsid (N), and non-structural protein chains (nsp1) and (nsp2) were most affected by the mutations. Specifically, amino acids D614G, N501Y, P681H, E484A, and P681R were observed to have significant effects on virus transmissibility, its ability to bind to host cells, and its ability to evade the immune response. These findings underscore the importance of understanding and monitoring these mutations to develop effective strategies for both the treatment and prevention of virus infection. With this patient-related epidemiological information approach and variant data, public health institutions in Ecuador can improve their understanding of disease dynamics, comprehend the importance of disease monitoring, and formulate health safety policies to prevent the recurrence of dangerous SARS-CoV-2 variants.

For future research, we propose incorporating more information from different reputable free databases to enhance the analysis and make comparisons with existing data. This may include integrating genomic and epidemiological information from various countries to identify global or specific patterns. We also suggest considering the inclusion of additional variables, such as environmental factors and the underlying medical conditions of the patients, to achieve a deeper and more holistic understanding of the infection.

Another crucial aspect would be to conduct a detailed analysis of the mutations identified in key amino acids. This would enable the evaluation of their individual impact on the virus’s interaction with host cells, their replicative capacity, and their influence on the immune response. The use of predictive models or machine learning algorithms to forecast the evolution of variants and their impact could also offer accurate early insight into possible disease scenarios.

## Figures and Tables

**Table 1:** Database variables, COVID-19, Ecuador.

Type of Data	Name	Type	Precision	Example
Genetic sequence	Protein chain	Text	Characters	[——–accaaccaactctaa.....]
Patient	Patient code	Text	Characters	EPI_ISL_10137512
	Length protein chain	Numeric	Integer	29,557
	Province	Text	Characters	Imbabura
	City	Text	Characters	Ibarra
	Latitude	Numeric	Integer with five decimal	0.35987
	Longitude	Numeric	Integer with five decimal	−78.12825
	Age	Text	Characters	18
	Gender	Text	Characters	Male
	Lineages	Text	Characters	BA.1.1
	Virus code	Text	Characters	VOI GRA
	Sampling date	Text	Characters	21 February 2022
	Variant	Text	Characters	Omicron

**Table 2:** Pearson correlation.

Variable	Age	Gender	Variant
Age	1	−0.31	0.85
Gender	−0.31	1	0.54
Variant	0.54	0.85	1

**Table 3:** Chi-square test.

Variable	Age	Gender	Variant
Age	-	6.84	185.08
Gender	6.84	-	89.98
Variant	185.08	89.98	-

**Table 4:** ANOVA method.

Variable	Age	Gender	Variant
Age	-	0.41	2.38
Gender	0.41	-	1.92
Variant	2.38	1.92	-

**Table 5:** Variants of SARS-CoV-2 across Ecuadorian provinces.

Variant	Province	Number of Infected	Infected (%)
Omicrom	Pichincha	2068	34.89
	Guayas	1132	19.10
	Azuay	385	6.50
	Cotopaxi	395	6.66
	Manabi	454	7.66
	Chimborazo	290	4.89
	Tungurahua	179	3.02
	Imbabura	131	2.21
	Santo Domingo de los Tsachilas	125	2.11
	Bolivar	148	2.50
	Province with less than 2%	620	10.46
	***Total***	***5927 ***	***100.00 ***
Delta	Guayas	261	18.64
	Pichincha	227	16.21
	Manabi	213	15.21
	El Oro	186	13.29
	Santo Domingo de Los Tsachilas	84	6.00
	Sucumbios	58	4.14
	Azuay	53	3.79
	Napo	46	3.29
	Los Rios	41	2.93
	Tungurahua	41	2.93
	Imbabura	30	2.14
	Province with less than 2%	160	11.43
	***Total ***	***1400 ***	***100.00 ***
Ex voi mu	Pichincha	198	31.83
	Guayas	91	14.63
	Chimborazo	87	13.99
	Cotopaxi	21	3.38
	Manabi	41	6.59
	Santo Domingo de los Tsachilas	34	5.47
	El Oro	28	4.50
	Los Rios	18	2.89
	Azuay	17	2.73
	Morona Santiago	15	2.41
	Imbabura	14	2.25
	Carchi	13	2.09
	Province with less than 2%	45	7.24
	***Total***	***622***	***100.00***
Gamma	Pichincha	112	29.32
	Guayas	77	20.16
	El Oro	25	6.55
	Chimborazo	21	5.50
	Azuay	21	5.50
	Manabi	22	5.76
	Cotopaxi	10	2.62
	Santo Domingo de los Tsachilas	12	3.14
	Carchi	12	3.14
	Imbabura	11	2.88
	Morona Santiago	11	2.88
	Tungurahua	8	2.09
	Sucumbios	9	2.36
	Province with less than 2%	31	8.12
	***Total ***	***382 ***	***100.00 ***
Ex Voi Lambda	Guayas	69	20.06
	El Oro	65	18.90
	Chimborazo	28	8.14
	Manabi	36	10.47
	Pichincha	53	15.41
	Los Rios	12	3.49
	Morona Santiago	18	5.23
	Azuay	9	2.62
	Imbabura	9	2.62
	Carchi	8	2.33
	Napo	8	2.33
	Province with less than 2%	29	8.43
	***Total ***	***344 ***	***100.00 ***
Alpha	Pichincha	124	39.24
	Chimborazo	48	15.19
	Guayas	32	10.13
	Santo Domingo de Los Tsachilas	17	5.38
	Los Rios	15	4.75
	Manabi	13	4.11
	Bolivar	7	2.22
	Loja	9	2.85
	Morona Santiago	9	2.85
	Province with less than 2%	42	13.29
	***Total ***	***316 ***	***100.00 ***

**Table 6:** Infection numbers and rates in Ecuadorian provinces grouped by SARS-CoV-2 variants.

Province	Variant	Number of Infected	Infected %
Pichincha	Omicron	2068	74.34
	Delta	227	8.16
	Ex Voi Mu	198	7.12
	Alpha	124	4.46
	Gamma	112	4.03
	Lambda	53	1.91
**Total **		***2782 ***	***100.00 ***
Guayas	Omicron	1132	68.11
	Delta	261	15.70
	Ex Voi Mu	91	5.48
	Gamma	77	4.63
	Lambda	69	4.15
	Alpha	32	1.93
***Total ***		***1662 ***	***100.00 ***
Manabi	Omicrom	454	58.28
	Delta	213	27.34
	Ex Voi Mu	41	5.26
	Lambda	36	4.62
	Gamma	22	2.82
	Alpha	13	1.67
***Total***		***779***	***100.00***
Chimborazo	Omicron	290	58.35
	Ex Voi Mu	87	17.51
	Alpha	48	9.66
	Lambda	28	5.63
	Delta	23	4.63
	Gamma	21	4.23
***Total***		***497***	***100.00***
Azuay	Omicron	385	78.41
	Delta	53	10.79
	Gamma	21	4.28
	Ex Voi Mu	17	3.46
	Lambda	9	1.83
	Alpha	6	1.22
***Total***		***491***	***100.00***
Cotopaxi	Omicron	395	88.37
	Ex Voi Mu	21	4.70
	Delta	16	3.58
	Gamma	10	2.24
	Lambda	3	0.67
	Alpha	2	0.45
***Total***		***447***	***100.00***
El Oro	Delta	186	53.30
	Lambda	65	18.62
	Ex Voi Mu	28	8.02
	Gamma	25	7.16
	Omicron	42	12.03
	Alpha	3	0.86
***Total***		***349***	***100.00***
Santo Domingo de Los Tsachilas	Omicrom	125	45.13
	Delta	84	30.32
	Ex Voi Mu	34	12.27
	Alpha	17	6.14
	Gamma	12	4.33
	Lambda	5	1.81
***Total***		***277***	***100.00***
Tungurahua	Omicron	179	72.76
	Delta	41	16.67
	Ex Voi Mu	11	4.47
	Gamma	8	3.25
	Alpha	5	2.03
	Lambda	2	0.81
**Total**		246	100.00
Imbabura	Omicron	131	65.17
	Delta	30	14.93
	Ex Voi Mu	14	6.97
	Gamma	11	5.47
	Lambda	9	4.48
	Alpha	6	2.99
***Total***		***201***	***100.00***
Bolivar	Omicrom	148	80.00
	Delta	24	12.97
	Alpha	7	3.78
	Ex Voi Mu	4	2.16
	Lambda	1	0.54
	Gamma	1	0.54
***Total***		***185***	***100.00***
Napo	Omicrom	88	57.14
	Delta	46	29.87
	Gamma	7	4.55
	Lambda	8	5.19
	Ex Voi Mu	4	2.60
	Alpha	1	0.65
***Total***		***154***	***100.00***
Los Rios	Omicron	62	40.52
	Delta	41	26.80
	Ex Voi Mu	18	11.76
	Alpha	15	9.80
	Lambda	12	7.84
	Gamma	5	3.27
***Total***		***153***	***100.00***
Sucumbios	Omicrom	69	46.62
	Delta	58	39.19
	Gamma	9	6.08
	Ex Voi Mu	6	4.05
	Alpha	4	2.70
	Lambda	2	1.35
***Total***		***148***	***100.00***
Esmeraldas	Omicron	95	73.08
	Delta	14	10.77
	Ex Voi Mu	10	7.69
	Lambda	5	3.85
	Alpha	4	3.08
	Gamma	2	1.54
***Total***		***130***	***100.00***
Carchi	Omicrom	64	58.18
	Ex Voi Mu	13	11.82
	Gamma	12	10.91
	Delta	9	8.18
	Lambda	8	7.27
	Alpha	4	3.64
***Total***		***110***	***100.00***
Morona Santiago	Lambda	18	21.43
	Omicrom	18	21.43
	Ex Voi Mu	15	17.86
	Delta	13	15.48
	Gamma	11	13.10
	Alpha	9	10.71
***Total***		***84***	***100.00***
Cañar	Omicrom	62	75.61
	Delta	10	12.20
	Gamma	5	6.10
	Lambda	2	2.44
	Ex Voi Mu	2	2.44
	Alpha	1	1.22
**Total**		**82**	**100.00**
Loja	Omicron	40	70.18
	Alpha	9	15.79
	Delta	3	5.26
	Gamma	3	5.26
	Lambda	2	3.51
	Ex Voi Mu	0	0.00
***Total***		***57***	***100.00***
Provinces with less than 50 cases	Omicrom	80	50.96
	Delta	48	30.57
	Lambda	7	4.46
	Gamma	8	5.10
	Ex Voi Mu	8	5.10
	Alpha	6	3.82
**Total**		**157**	**100.00**

**Table 7:** Percentage of infected patients by age and variant.

Variant	26–30 Years (%)	31–35 Years (%)
Omicron	16.38	22.17
Delta	17.21	20.93
Mu Gh	15.11	19.45
Gamma	17.02	21.73
Lambda	17.15	15.7
Alpha	16.38	22.17

**Table 8:** Amino acids affected by two mutations.

Mutation	Protein	Position	Original Sequence	Mutated Sequence
DEL144/144	Not Assigned	144	-	-
DEL157/158		157/158	-	-
DEL241/243		241/243	-	-
DEL25/27		25/27	-	-
DEL3675/3677		3675/3677	-	-
DEL69/70		69/70	-	-
E1264D		1264	Glu (E)	Asp (D)
G662S	Spike (S)	662	Gly (G)	Ser (S)
I1566V		1566	Ile (I)	Val (V)
I2230T		2230	Ile (I)	Thr (T)
K3353R		3353	Lys (K)	Arg (R)
P1000L		1000	Pro (P)	Leu (L)
P2046L		2046	Pro (P)	Leu (L)
P2287S		2287	Pro (P)	Ser (S)
P3395H		3395	Pro (P)	His (H)
S1188L		1188	Ser (S)	Leu (L)
T1001I		1001	Thr (T)	Ile (I)
T265I		265	Thr (T)	Ile (I)
T3255I		3255	Thr (T)	Ile (I)
T3646A		3646	Thr (T)	Ala (A)
V2930L		2930	Val (V)	Leu (L)
A1306S		1306	Ala (A)	Ser (S)
A1708D		1708	Ala (A)	Asp (D)
A1918V		1918	Ala (A)	Val (V)

**Table 9:** Amino acids affected by a mutation.

Mutation	Protein	Position	Original Sequence	Mutated Sequence
D614G	Spike(s)	614	Asp (D)	Gly (G)
S84L		84	Ser (S)	Leu (L)
P681H		681	Pro (P)	His (H)
H655Y		655	His (H)	Tyr (Y)
N679K		679	Asn (N)	Lys (K)
H69X		69	His (H)	Insertion X
D796Y		796	Asp (D)	Tyr (Y)
V70X		70	Val (V)	Insertion X
T478K		478	Thr (T)	Lys (K)
A63T		63	Ala (A)	Thr (T)
N501Y		501	Asn (N)	Tyr (Y)
S375F		375	Ser (S)	Phe (F)
S373P		373	Ser (S)	Pro (P)
G339D		339	Gly (G)	Asp (D)
T223I		223	Thr (T)	Ile (I)
S413R		413	Ser (S)	Arg (R)
Y505H		505	Tyr (Y)	His (H)
Q498R		498	Gln (Q)	Arg (R)
E484A		484	Glu (E)	Ala (A)
S477N		477	Ser (S)	Asn (N)
T376A		376	Thr (T)	Ala (A)
K417N		417	Lys (K)	Asn (N)
S371F		371	Ser (S)	Phe (F)
D405N		405	Asp (D)	Asn (N)
DEL31/33		31/33	Deletion	Deletion
L452R		452	Leu (L)	Arg (R)
R408S		408	Arg (R)	Ser (S)
N440K		440	Asn (N)	Lys (K)
R203K	Nucleocapsid (N)	203	Arg (R)	Lys (K)
G204R		204	Gly (G)	Arg (R)
N969K		969	Asn (N)	Lys (K)
Q954H		954	Gln (Q)	His (H)
N764K		764	Asn (N)	Lys (K)
T9I		9	Thr (T)	Ile (I)
Q19E	Non-structural (nsp2)	19	Gln (Q)	Glu (E)
G142D		142	Gly (G)	Asp (D)
T19I		19	Thr (T)	Ile (I)
Y144X		144	Tyr (Y)	Insertion X
T95I		95	Thr (T)	Ile (I)
D3N		3	As	-
P13L	Non-structural (nsp1)	13	Pro (P)	Leu (L)

**Table 10:** Amino acids affected by two mutations in different variants.

Mutation	Variants	Protein
T265I	Omicron, Delta, Lambda, Mu GH, Gamma, Alpha	Spike (S)
P3395H	Omicron, Delta, Lambda, Mu GH, Alpha	Spike (S)
S1188L	Delta, Lambda, Mu GH, Gamma, Alpha	Spike (S)
A1306S	Omicron, Lambda, Mu GH, Alpha	Spike (S)
A1708D	Omicron, Lambda, Mu GH, Alpha	Spike (S)
A1918V	Omicron, Delta, Mu GH, Alpha	Spike (S)
DEL144/144	Omicron, Delta, Mu GH, Alpha	Spike (S)
DEL157/158	Omicron, Delta, Mu GH, Alpha	Spike (S)
DEL241/243	Omicron, Delta, Mu GH, Alpha	Spike (S)
DEL25/27	Omicron, Delta, Mu GH, Alpha	Spike (S)
DEL69/70	Omicron, Delta, Mu GH, Alpha	Spike (S)
E1264D	Omicron, Delta, Mu GH, Alpha	Spike (S)
G662S	Omicron, Mu GH, Alpha	Spike (S)
I1566V	Omicron, Delta, Mu GH, Alpha	Spike (S)
I2230T	Omicron, Lambda, Mu GH, Alpha	Nonstructural (nsp2)
K3353R	Omicron, Lambda, Mu GH, Alpha	Nonstructural (nsp2)
P1000L	Omicron, Delta, Mu GH, Alpha	Spike (S)
P2287S	Omicron, Lambda, Mu GH, Alpha	Nonstructural (nsp2)
T1001I	Lambda, Mu GH, Gamma, Alpha	Spike (S)
T3255I	Omicron, Delta, Lambda, Mu GH	Nonstructural (nsp2)
T3646A	Omicron, Delta, Lambda, Mu GH, Alpha	Nonstructural (nsp2)
V2930L	Omicron, Lambda, Mu GH, Alpha	Nonstructural (nsp2)
L24S	Mu GH, Alpha	c
P26S	Mu GH, Alpha	Nonstructural (nsp1)
P681R	Mu GH, Alpha	Spike (S)
T19R	Mu GH, Alpha	Spike (S)
E484A	Mu GH	Nucleocapsid (N)
K1655N	Gamma	Nucleocapsid (N)
K1795Q	Gamma	Nucleocapsid (N)
L18F	Gamma	Spike (S)
R346T	Mu GH	Nucleocapsid (N)
T19I	Mu GH	Nonstructural (nsp1)
T20N	Gamma	Spike (S)
Y144X	Mu GH	Spike (S)

**Table 11:** Amino acids affected by a mutation in different variants.

Number	Amino Acid	Variants	Proteins
1	D614G	Alpha, Mu GH, Lambda, Delta, Omicron	Spike (S)
2	N501Y	Alpha, Mu GH, Omicron	
3	P681H	Alpha, Mu GH, Omicron	
4	DEL31/33	Alpha, Mu GH, Lambda, Delta	
5	S84L	Alpha, Mu GH, Lambda, Delta, Omicron	
6	R203K	Alpha, Mu GH, Lambda, Omicron	
7	G204R	Alpha, Mu GH, Lambda, Omicron	
8	T40I	Alpha, Lambda, Delta, Omicron	Not defined

## Data Availability

The data are available on the GISAID site: https://gisaid.org/, (accessed on 1 December 2023 ).
